# ATR-FTIR, EDS and SEM evaluations of enamel structure after treatment with hydrogen peroxide bleaching agents loaded with nano-hydroxyapatite particles

**DOI:** 10.7717/peerj.10606

**Published:** 2021-01-29

**Authors:** Giulia Orilisi, Vincenzo Tosco, Riccardo Monterubbianesi, Valentina Notarstefano, Mutlu Özcan, Angelo Putignano, Giovanna Orsini

**Affiliations:** 1 Department of Clinical Sciences and Stomatology, Polytechnic University of Marche, Ancona, Italy; 2 Department of Life and Environmental Sciences, Polytechnic University of Marche, Ancona, Italy; 3 Division of Dental Biomaterials, Center for Dental and Oral Medicine, Clinic for Reconstructive Dentistry, University of Zurich, Zurich, Switzerland

**Keywords:** SEM, ATR-FTIR spectroscopy, Hydrogen peroxide, Bleaching, Carbamide peroxide, Nano-hydroxyapatite

## Abstract

**Background:**

Tooth whitening is one of the most requested dental treatments, but it still presents some side effects. Indeed, the bleaching agent can generate patients’ discomfort and dental hard tissue damages, not achieving an efficient and long-lasting treatment with optimum whitening effect. To overcome these limitations, the bleaching agents containing nano-hydroxyapatite can represent a reliable solution to avoid these detrimental effects.

**Methods:**

In this study, human third molars were treated with commercial bleaching agents, containing nano-hydroxyapatite (nHA) and 6% (at-home treatment), 12% and 18% (in-office treatments) of hydrogen peroxide (HP), named respectively G1, G2 and G3. The results were evaluated descriptively and analytically using Attenuated Total Reflectance-Fourier Transform Infrared Spectroscopy (ATR-FTIR), Scanning Electron Microscopy (SEM) and Energy Dispersive X-ray spectroscopy (EDS), comparing the treated groups with a commercial gel containing 10% carbamide peroxide (CONV) and with a non-treated control group (CTRL).

**Results:**

ATR-FTIR analysis revealed a similar composition in carbonates and phosphates for CTRL, G1 and G2 groups, which showed no significant differences in their spectral profiles; an increased amount of organic matter was found in G3, while CONV displayed an intermediate behavior. SEM analysis did not highlight significant changes in the enamel microstructure of G1 and CONV when compared to CTRL; the pattern observed in G2 presented a slight increase of enamel irregularities, while G3 displayed a partial removal of the aprismatic layer and microporosities. No evident effects due to nHA were observed in the structure of the hydroxyapatite component of G1, G2 and CONV, if compared to CTRL, while G3 showed a slight loss of crystallinity. In all groups, EDS identified slight changes in the concentration of chemical elements O and Ca, while the Ca/P ratio was similar when compared to CTRL.

**Conclusion:**

The obtained results suggest that the application of the tested commercial bleaching agents, with a concentration of HP up to 12%, does not alter the morphological and chemical composition of the enamel surface and maintains its crystallinity.

## Introduction

In the last decades, patients’ demand for aesthetic dental procedures, including bleaching, has considerably risen and, hence, tooth whitening has become an important topic in dentistry (Demarco, Meireles & Masotti, 2009).

Tooth color depends on both intrinsic and extrinsic stains (Dabanoglu et al., 2009). Intrinsic discolorations are caused by the incorporation of chromophores into dentin and enamel during odontogenesis or after an eruption, for example, hereditary disorders, tetracycline medication, excessive fluoride uptake or high fever associated with early childhood diseases (Dabanoglu et al., 2009; Sherwood, 2010). Extrinsic discolorations are the result of the adhesion on the tooth surface of chromophores, contained, for example, in coffee, tea, red wine, carrots, oranges, or tobacco. Since 1864, to avoid this inconvenience, whitening formulations, both at home and in-office, have been developed in dental practice. While most external stains can be removed by a thorough tooth cleaning, for correcting or at least reducing intrinsic discoloration and discoloration resistant to cleaning, the application of oxidizing agents, which directly break the double bonds of chromophores, is strongly required (Watts & Addy, 2001; Dahl & Pallesen, 2003; Jin et al., 2013). This procedure, referred to as bleaching, produces smaller molecules that absorb light with a shorter wavelength than visible light (such as ultraviolet light), and hence the teeth no longer appear to be colored (Dadoun & Bartlett, 2003; Dabanoglu et al., 2009).

Despite its popularity, there are severe side effects associated with the use of chemical oxidants, such as gingival irritation, modifications in surface morphology and tooth hypersensitivity (Pintado-Palomino et al., 2015; Rodríguez-Martínez, Valiente & Sánchez-Martín, 2019). The enamel structure plays an important role in the wellbeing of dental hard tissues; therefore, it is important to preserve it, even after treatments with bleaching agents (Orilisi et al., 2020). It is accepted that, under supervised clinical conditions, dental bleaching is safe for enamel integrity, but less is known about abusive and/or over-the-counter use (Joiner et al., 2008; Demarco, Meireles & Masotti, 2009; Joiner, 2010; Silveira et al., 2018). Some researchers have evaluated the effects of aggressive bleaching treatments on tooth structure, observing changes in the surface integrity and on the microstructure of enamel crystals, and a higher susceptibility to demineralization (Cavalli et al., 2004; Shi et al., 2012). Concerning the in-office products, they are commonly comprised of different concentrations of peroxide, varying from 5 to 40% of hydrogen peroxide (HP) or carbamide peroxide (CP) (Grazioli et al., 2018). It is believed that HP penetrates enamel and dentin and, due to its chemical instability, it is decomposed into different active oxygen species under specific temperature, pH and light conditions (Kwon & Wertz, 2015); these free radicals may oxidize the conjugated chain of the chromophores (Kwon & Wertz, 2015), and, furthermore, the oxidation could promote morphological changes of the enamel (Kawamoto & Tsujimoto, 2004). On the other hand, CP dissociates into HP (approximately a third of its former concentration) and urea, which further breaks down into water and ammonia (Da Silva Marques et al., 2012; Da Costa et al., 2012). It has been ascertained that the risk of adverse effects increases with the peroxide concentration (Perdigão, Baratieri & Arcari, 2004). According to this finding, it is still necessary to develop a more effective, efficient, and long-lasting treatment to achieve an optimum whitening effect without any damage.

One of the last frontiers to overcome the demineralization is represented by the clinical application of nano-hydroxyapatite (nHA), within different carriers. Hydroxyapatite (HA) is a calcium phosphate compound with the molecular formula Ca_10_ (PO_4_) OH_2_ and a calcium-to-phosphorus ratio of 1:67. There are several forms of calcium phosphate in nature, but the most stable is represented by HA (Bordea et al., 2020). It can be considered as a revolutionary material due to its remarkable remineralizing effects on enamel initial lesions, it performs a protective action against caries and dental erosion (Huang, Gao & Yu, 2009). The nHA presents a strong affinity with demineralized surfaces due to its ability to bind to pores created by acid attacks. Indeed, after adhering, nHA multiplies and organizes into microclusters to form a uniform apatite layer that may completely overlap interprismatic and prismatic enamel (Swarup & Rao, 2012). According to these findings, in order to prevent hypersensitivity after bleaching, which can occur in a 70% of bleached patients, whitening gel has been enriched with HA in its nano form (Kutuk et al., 2018).

Therefore, this in vitro study investigates the effects on the enamel surface of the following three new commercial agents containing nHA and increasing amounts of HP: one at-home gel based on 6% HP, and two in-office ones, available at different concentrations of HP (12% and 18%). The effects of their application on human tooth surfaces were compared with the ones displayed by a commercially available CP-based, at-home bleaching gel (CONV). A non-treated group was also analyzed as control (CTRL). Surface enamel morphologies of all groups were qualitatively evaluated by Scanning Electron Microscopy (SEM), whereas Attenuated Total Reflectance-Fourier Transform Infrared Spectroscopy (ATR-FTIR) and Energy Dispersive X-ray Spectroscopy (EDS) were used to investigate respectively the effects of these bleaching agents on enamel chemical structure and elemental composition of the tooth surface, focusing on changes in the composition of Ca and P. The coupling of these analytical techniques represents a valuable tool to obtain a comprehensive overview of the morphological and chemical alterations of tooth surfaces. FTIR spectroscopy is a well-assessed method for studying the structural features in biomaterials (Paschalis, Mendelsohn & Boskey, 2011; Kowalczuk & Pitucha, 2019). By the analysis of the spectral profile and specific band area ratios, it is possible to evaluate the chemical composition of enamel and dentine and relate it to external treatments and pathologies (Verdelis et al., 2007; Lubarsky et al., 2012; Liu et al., 2014). EDS determines the mineral content of dental hard tissues. The main advantage of this system is its capability to provide an accurate and non-destructive analysis of the specimens (Furlan et al., 2017; Llena, Esteve & Forner, 2018; Kutuk et al., 2018). As a consequence, this method was used to evaluate the changes in the mineral content of enamel. SEM analysis allows the visualization of images at high magnification and, hence, it is a useful approach for qualitative analysis of the enamel morphology (Wang et al., 2013).

The following two null hypotheses were tested: (1) the three bleaching agents based on HP and nHA do not affect the morphology and chemical composition of the enamel surface and display features similar to the CP-based agent (CONV); (2) the three bleaching agents based on HP and nHA do not affect the enamel structure.

## Materials and Methods

### Teeth collection and samples preparation

Ten sound extracted third molars were collected, from subjects aged between 18 and 30 years (Sulieman et al., 2004; Coceska et al., 2016). Teeth were surgically extracted for orthodontic reasons at the Section of Stomatology of DiSCO Department, Polytechnic University of Marche, Ancona, Italy. According to the Local Ethics Committee guidelines and the 1964 Helsinki Declaration, informed consent was obtained from the subjects that were aware that their hard dental tissues, as discard of the surgical procedures, would be used for research purposes. After the surgical extraction, teeth were washed in an ultrasonic bath with distilled water for 2 min, in order to remove blood and biological remains, and then carefully examined to exclude the presence of lesions and decays, including hypoplastic defects and cracks: tooth exhibiting any of these features were excluded. Selected teeth were stored in a 0.5% (w/w) chloramine solution (NH_2_Cl); immediately before the beginning of the application of the whitening products, they were cut, using a diamond saw (Buehler Isomet 1000, USA) with copious water irrigation, in mesiodistal direction to separate the buccal and lingual surfaces, starting at the level of the occlusal fissure.

### Bleaching treatments

**Figure 1:**
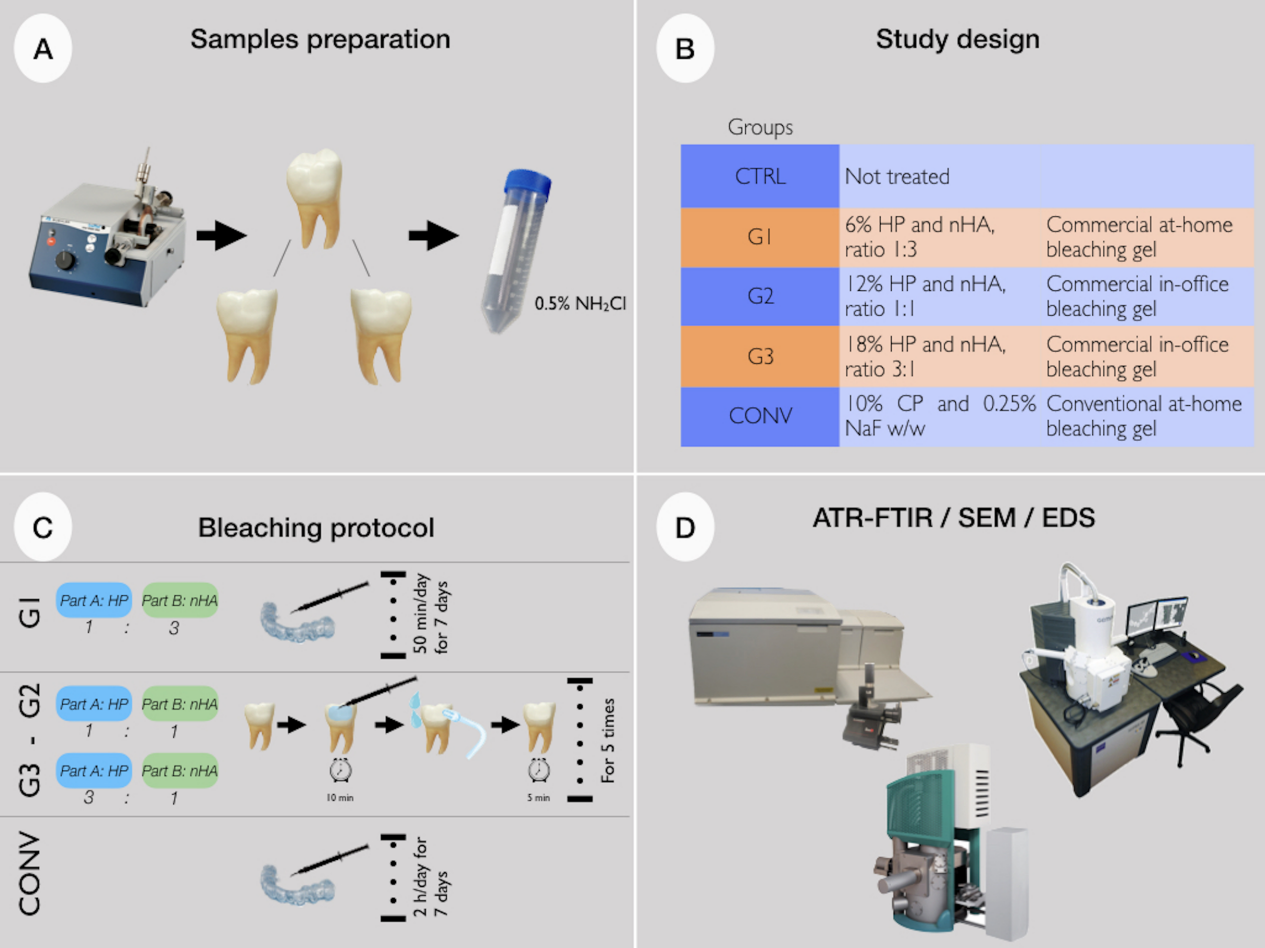
(A) Teeth samples preparation; (B) study design; (C) bleaching protocols; (D) ATR-FTIR spectroscopy, SEM microscopy and EDS evaluations. HP, hydrogen peroxide; CP, carbamide peroxide; nHA, nano-hydroxyapatite; ATR-FTIR, attenuated total reflectance-fourier transform infrared spectroscopy; SEM, scanning electron microscope; EDS, energy dispersive X-ray spectroscopy.

The following bleaching agents were in vitro tested: G1, at-home gel based on 6% HP (part A) and nano-hydroxyapatite (nHA, part B) in a ratio 1:3 (BioWhiten, Biodent Ltd., Istanbul, Turkey); G2, in-office gel based on 12% HP (part A) and nHA (part B) in a ratio 1:1 (BioWhiten, Biodent Ltd., Istanbul, Turkey); G3, in-office gel based on 18% HP (part A) and nHA (part B) in a ratio 3:1 (BioWhiten, Biodent Ltd., Istanbul, Turkey); CONV, commercially available at-home gel based on 10% CP (White Dental Beauty, Novon, Optident Optident, Ilkley, West Yorkshire). Part A and Part B of G1, G2 and G3 were mixed before application to obtain the above indicated HP and nHA ratios. Details about the bleaching agents (in terms of manufacturer’s instructions and chemical composition) as well as the precise exposure times are given in Table 1. Teeth portions were randomly divided into five groups (*n* = 3 for each group) and submitted to the following treatments: CTRL (Control Group; untreated teeth); G1 (teeth treated with the G1 bleaching gel for 50 min/day for 7 days); G2 (teeth treated with the G2 bleaching gel for 10 min for five times); G3 (teeth treated with the G3 bleaching gel for 10 min for five times); CONV (teeth treated with CONV bleaching gel for 2 h/day for 7 days). Each bleaching treatment was performed according to the manufacturer’s instructions. After each treatment, the bleaching agents were removed using a saliva aspirator and thoroughly rinsing the sample with distilled water. Between one application and another, samples were stored in distilled water at room temperature. In Fig. 1A a sketch of the experimental design is reported. All tooth samples were analysed by ATR-FTIR, SEM and EDS.

**Table 1:** Teeth experimental groups and chemical composition of bleaching agents.

Experimental groups	Manufacturer	Chemical composition	Exposure time
CTRL	None	None	None
G1	BioWhiten, Biodent Ltd., Istanbul, Turkey	Water, Glycerin, Alcohol, Sodium bicarbonate, Sodium hydroxide, 6% HP and nHA* (ratio 1:3)	50 min/day for 7 days
G2	BioWhiten, Biodent Ltd., Istanbul, Turkey	Water, Glycerin, Alcohol, Sodium bicarbonate, Sodium hydroxide, 12% HP and nHA (ratio 1:1)	10 min for 5 times
G3	BioWhiten, Biodent Ltd., Istanbul, Turkey	Water, Glycerin, Alcohol, Sodium bicarbonate, Sodium hydroxide, 18% HP and nHA* (ratio 3:1)	10 min for 5 times
CONV	White Dental Beauty, Novon, Optident Ilkley, West Yorkshire	10% CP and 0.25% w/w NaF (10% CP = ~3.3% HP)	2 h/day for 7 days

**Note:**

* nHA morphology; rod-like, width 5–20 nm (typically close to 10 nm) and length < 50 nm (typically between 20 and 40 nm).

### ATR-FTIR spectroscopy

Attenuated Total Reflectance-Fourier Transform Infrared Spectroscopy measurements were carried out by using a Perkin Elmer Spectrum GX1 spectrophotometer, equipped with a diamond/ZnSe Attenuated Total Reflection (ATR) accessory. Teeth were accurately positioned on the crystal surface in order to obtain a good contact between the crystal itself and the external side of each tooth (not on the side exposed to cutting). On each sample, ten IR spectra were collected in the spectral range 4,000–650 cm^−1^, with a spectral resolution of 4 cm^−1^; each spectrum was the average of 64 scans. Raw IR spectra were converted in absorbance mode, 13-points smoothed, two-points baseline linear fitted, and then vector normalized in the entire spectral range (OPUS 7.1, Bruker Optics, Ettlingen, Germany). For each tooth portion, the average IR spectrum was calculated, together with the average ± standard deviation spectra, which were used to evaluate the homogeneity of sample spectra (OPUS 7.1). Average IR spectra were interpolated in the 1,800–650 cm^−1^ range, two-points baseline linear fitted, and then curve fitted by using the GRAMS/AI 9.1 software (Galactic Industries, Inc., Salem, NH, USA); a Gaussian algorithm was adopted. The center of the subcomponent bands was imposed based on second derivative minima positions (wavenumbers, cm^−1^). To evaluate the goodness of the fitting, the spectral residual (obtained as the difference between the original trace and the fitted trace) was checked: fitting was considered satisfactory when this parameter was near to zero. For each subcomponent band, the position (in terms of wavenumbers, cm^−1^), the integrated Area (A), and the Full Width at Half Maximum (FWHM) were determined. The assignment of IR peaks was performed according to literature (Gadaleta et al., 1996; Boskey & Mendelsohn, 2005). Specific band area ratios were calculated.

### Scanning Electron Microscopy

Scanning Electron Microscopy observations were carried out by a Zeiss Supra 40 field-emission electron microscope (Polytechnic University of Marche, Department of Materials, Environmental Science and Urban Planning (SIMAU)). The same samples analysed by ATR-FTIR, a non-destroying analytical technique, were assembled in a sample holder and metallized with vacuum precipitation of a gold film on the dental surface. Scanning electron micrographs of the enamel were obtained with magnifications of 400× and 2,000×. SEM operated at 30 kV and at a 12 mm working distance. The obtained micrographs were evaluated descriptively, observing the variations in the micromorphology of the dental enamel of all different analysed groups.

### Energy Dispersive X-ray Spectroscopy

The enamel chemical characterization of the samples was carried out by EDS, performed by the Scanning Electron Microscope equipped with microanalysis EDAX Element (TESCAN VEGA3). The same samples were used for SEM and EDS. The operating parameters were: 15 mm working distance, 25 kV accelerating voltage, and magnification of 400×. The concentrations by weight (%) of the following chemical elements were evaluated: Oxygen (O), Fluorine (F), Sodium (Na), Phosphorous (P), Chlorine (Cl) and Calcium (Ca).

### Statistical analysis

Data deriving from ATR-FTIR and EDS analyses were presented as mean ± standard deviation (SD). Significant differences between experimental groups were determined by means of Kruskal–Wallis test, followed by Dunn’s test, using the statistical software package Prism6 (GraphPad Software, Inc., San Diego, CA, USA). The group size was set at *N* = 3 for all the experimental groups; significance was set at *p* < 0.05. Adjusted *p* values were calculated for each multiple comparison and reported in Tables S1 and S2.

## Results

**Figure 2:**
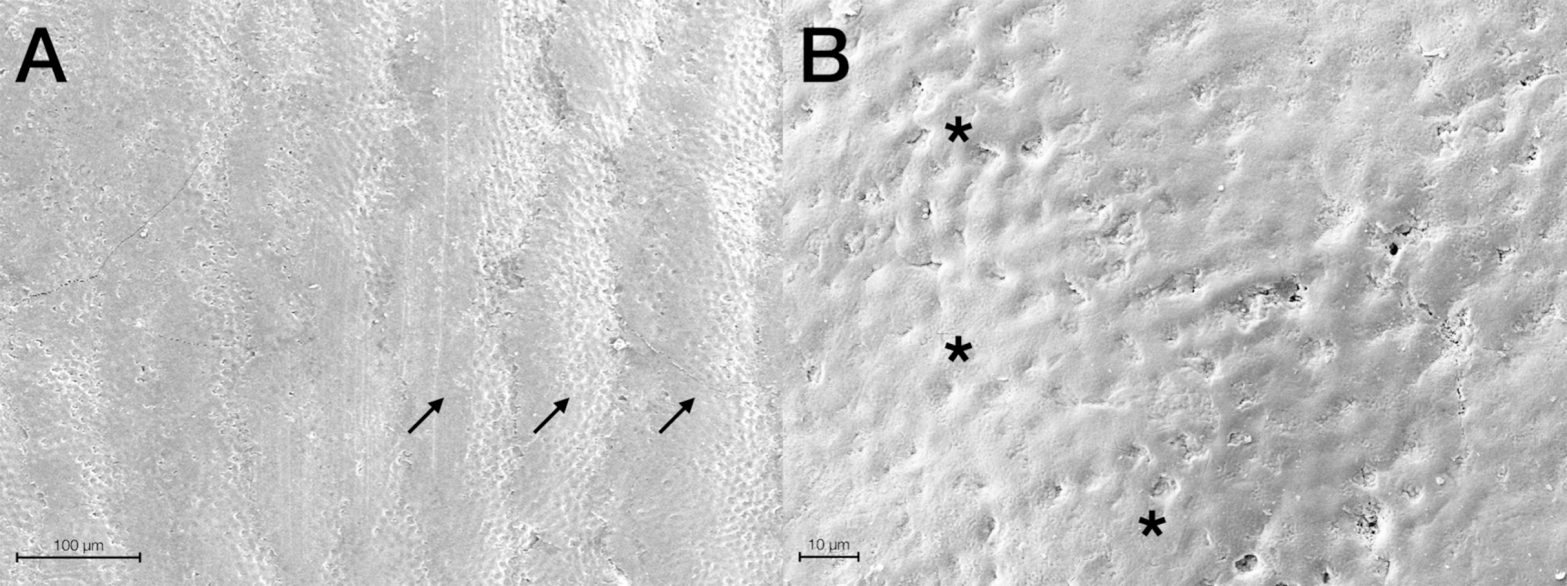
Scanning electron micrographs of a representative CTRL tooth sample at (A) 400× and (B) 2,000× original magnification. The micrograph A presents the typical aspect of sound enamel morphology, with enamel-like crystal structure and perikymata (black arrows). The micrographs B displays a homogeneous smooth area between the interprismatic rods (asterisks).

**Figure 3:**
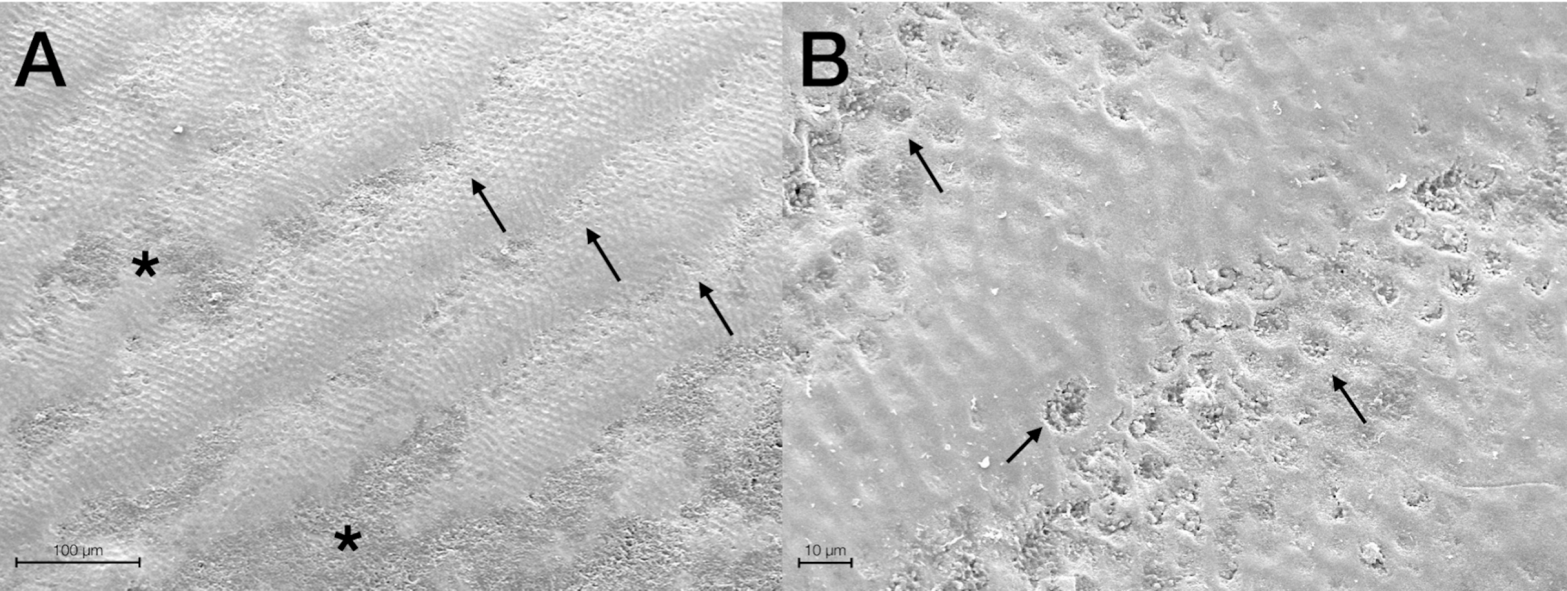
Scanning electron micrographs of a representative G1 tooth sample at (A) 400× and (B) 2,000× original magnification. The micrograph A shows a sound enamel morphology with enamel-like crystal structure and perikymata (black arrows), within minimal loss of enamel surface integrity (black asterisks). The micrograph B highlights well-preserved interprismatic rods and a slight dissolution of prism cores (black arrows).

**Figure 4:**
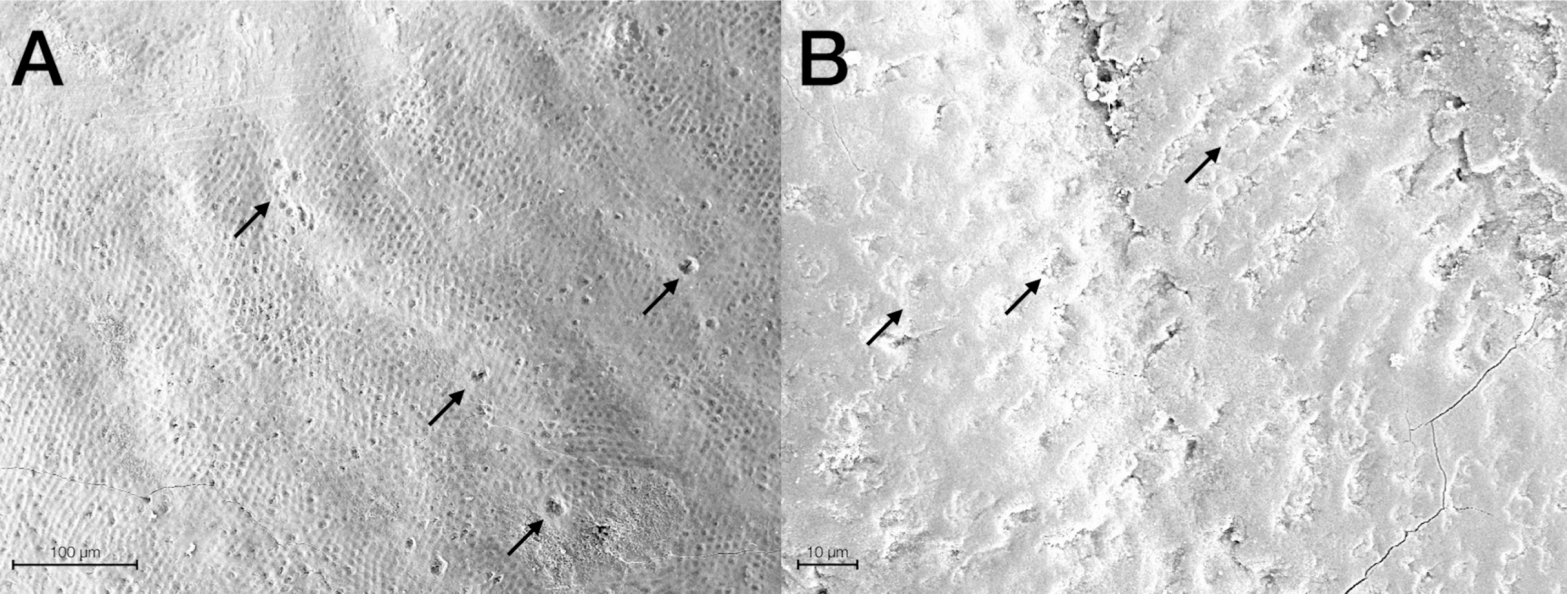
Scanning electron micrographs of a representative G2 tooth sample at (A) 400× and (B) 2,000× original magnification. The micrograph A presents a minimal loss of surface integrity with the presence of micropores (black arrows), partial loss of perikymata and raised enamel interrods. The micrograph B highlights the partial core prisms dissolution (black arrows) by the raised interrod structure.

**Figure 5:**
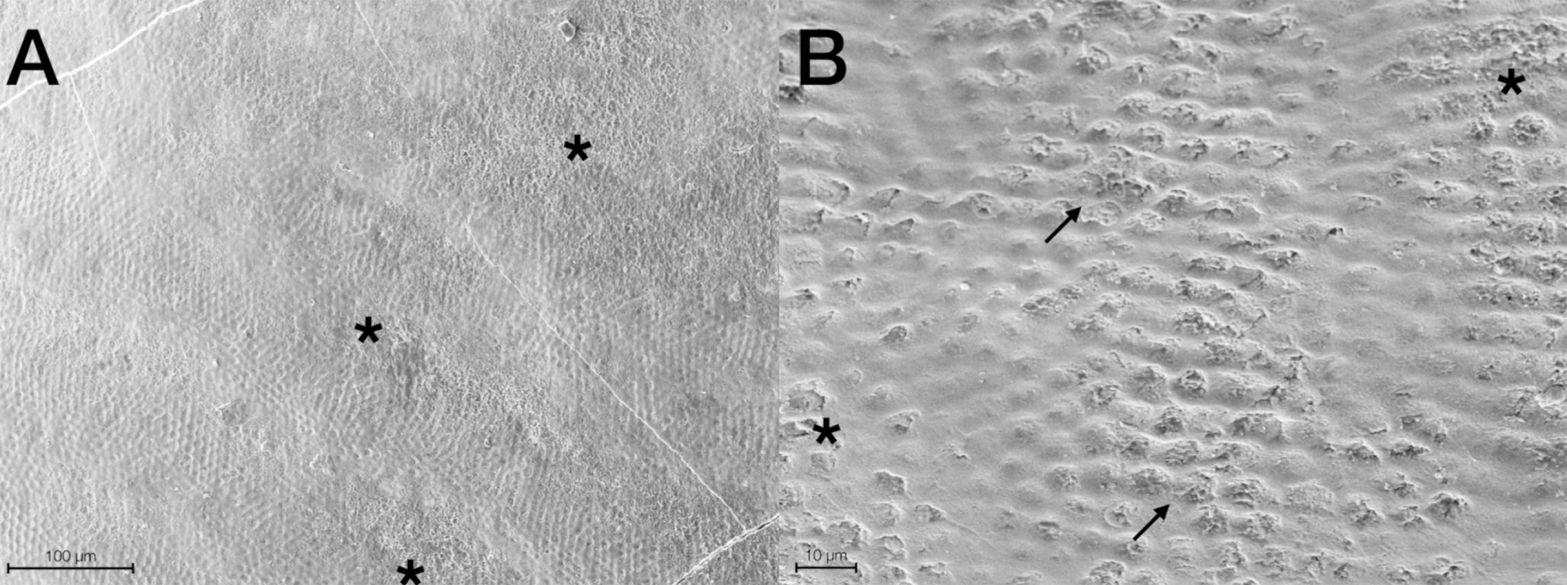
Scanning electron micrographs of a representative G3 tooth sample at (A) 400× and (B) 2,000× original magnification. The micrograph A displays a partial loss of surface integrity with some enamel micro-erosion (black asterisks), loss of perikymata and raised interrods. The micrograph B shows different enamel micro-erosions (black asterisks), associated with partial prism core dissolution and slight disorganization of interprismatic rods structure (black arrows).

**Figure 6:**
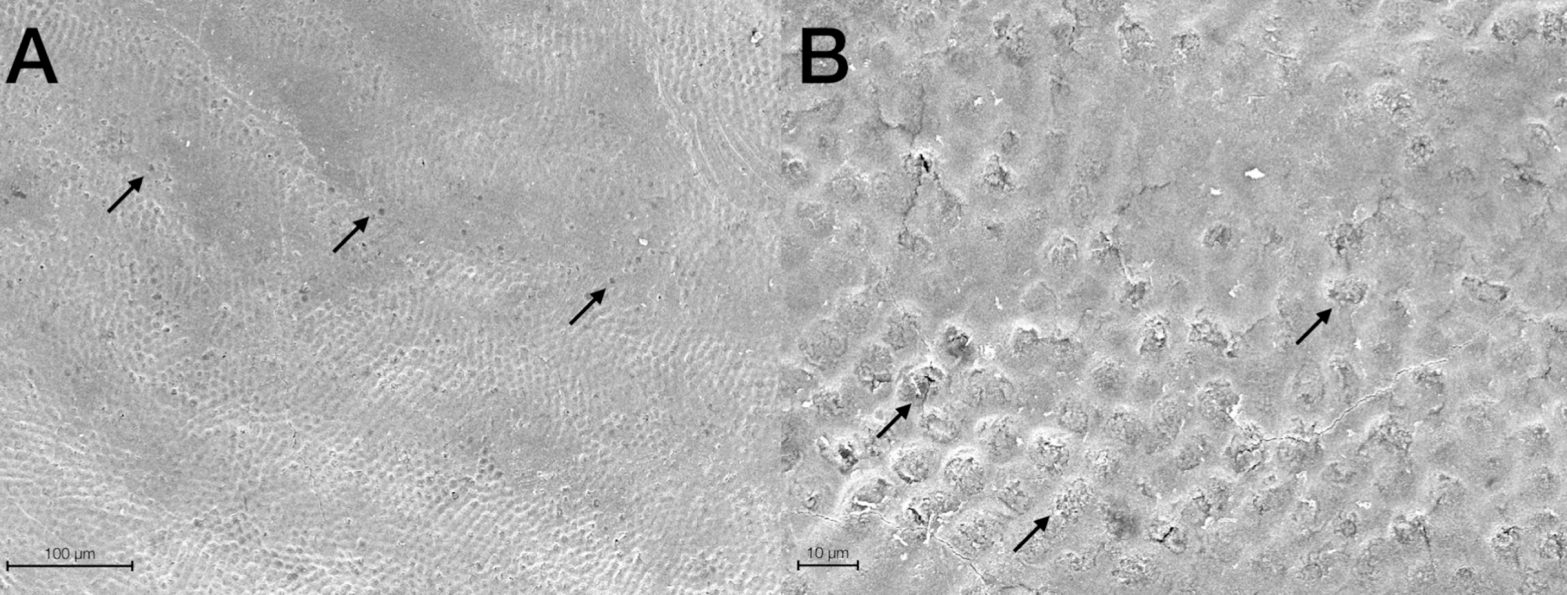
Scanning electron micrographs of a representative CONV tooth sample at (A) 400× and (B) 2,000× original magnification. The micrograph A shows a sound enamel morphology, with enamel-like crystal structures and minimal loss of surface integrity; some micropores (black arrows), loss of perikymata and raised enamel interrods are also identified. The micrograph B highlights well-preserved interprismatic rods, although minimal enamel erosion and slight prism cores dissolution (black arrows) are present.

Scanning Electron Microscopy analysis was exploited to investigate and compare the surface morphology of each treated group with that of untreated one (CTRL). Figures 2–6 showed the scanning electron micrographs of the enamel surface of representative tooth samples subjected to the different bleaching treatments proposed. Although at low magnification (400×), all micrographs of each bleached group showed apparently intact surfaces, similar to those of CTRL, at high magnification (2,000×) differences among groups were observed. In Figs. 2A and 2B, the micrographs of a representative CTRL tooth sample are reported; although the surface is not completely smoothened, the presence of aprismatic enamel and perikymata can be observed, suggesting that the physiological structure is preserved. Conversely, in all teeth samples submitted to bleaching (Figs. 3–6), signs of the treatments were randomly observed on the enamel surface, even though different types of defects and distinct severity of such events were found. Indeed, all G1 samples displayed a sound and intact enamel surface similar to that of CTRL, except one, which revealed a minimal loss of integrity, with an increase of enamel irregularities (Figs. 3A and 3B). Also, in G2, most scanning electron micrographs showed slight changes on the surface, with the presence of microporosities, but only in one sample, at higher magnification, an irregular surface pattern, with partial dissolution of the prisms, was observed (Figs. 4A and 4B). With the increase in the percentage of HP, morphological surface alterations became much more pronounced. In fact, changes in the enamel bleached surface were well evident in G3; in this group, the same pattern of alterations already observed in G2 was found, but in a more severe manner, together with an increased number of microporosities on the enamel surface (Figs. 5A and 5B). Moreover, in G3, the dissolution of prism cores was often present, together with partial micro-erosions in the surface (Fig. 5B). In CONV minimal defects, similar to those found in G1 samples, were detected. In particular, the micrographs showed a slight increase of irregularities, with a partial loss of the enamel surface integrity, and a little occurrence of microporosities (Figs. 6A and 6B).

**Figure 7:**
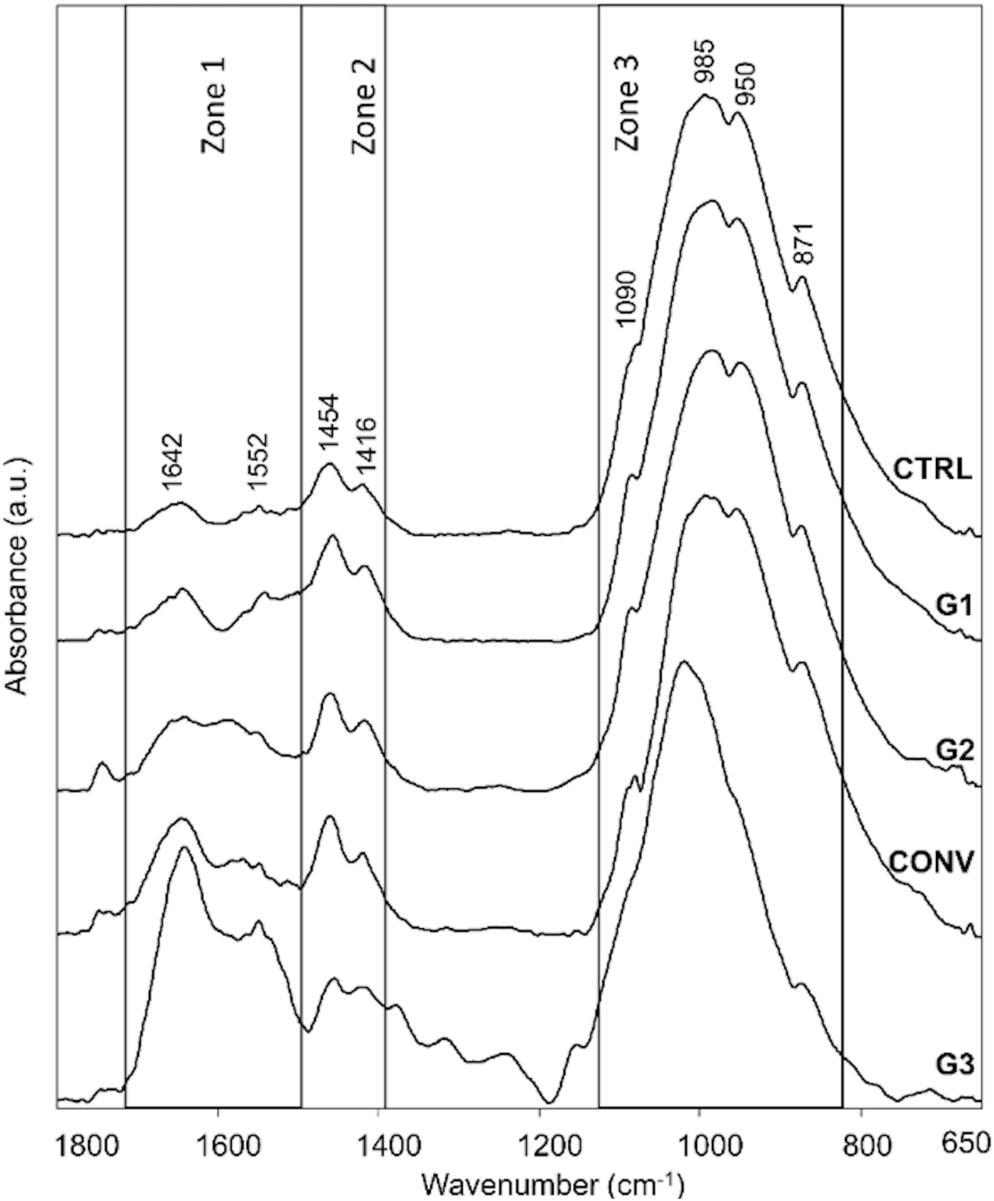
Average IR spectra of CTRL, G1, G2, CONV and G3 teeth samples. Spectra are showed in the 1,800–650 cm^−1^ spectral range and are shifted along *y*-axis for a better understanding of the figure. The position of the most meaningful IR bands of CTRL samples is reported in terms of wavenumbers (cm^−1^). Black boxes indicate the spectral ranges related to the organic (Zone 1) and inorganic (carbonates, Zone 2 and phosphates, Zone 3) matrices.

**Figure 8:**
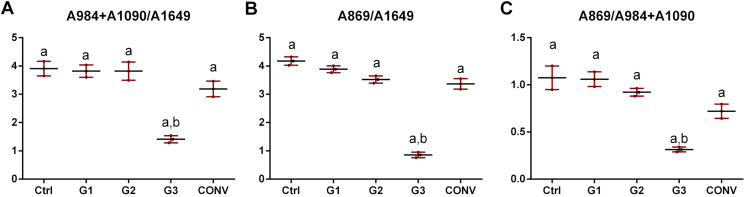
Statistical analysis of the following band area ratios. (A) A984+A1090/A1649 (ν1+n3 PO_4_/Amide I); (B) A869/A1649 (nCO_3_/Amide I) and (C) A869/A984+A1090 (nCO_3_/ν1+n3 PO_4_). Data are reported as mean ± S.D. Different letters indicate statistically significant differences among groups (*p* < 0.05).

**Figure 9:**
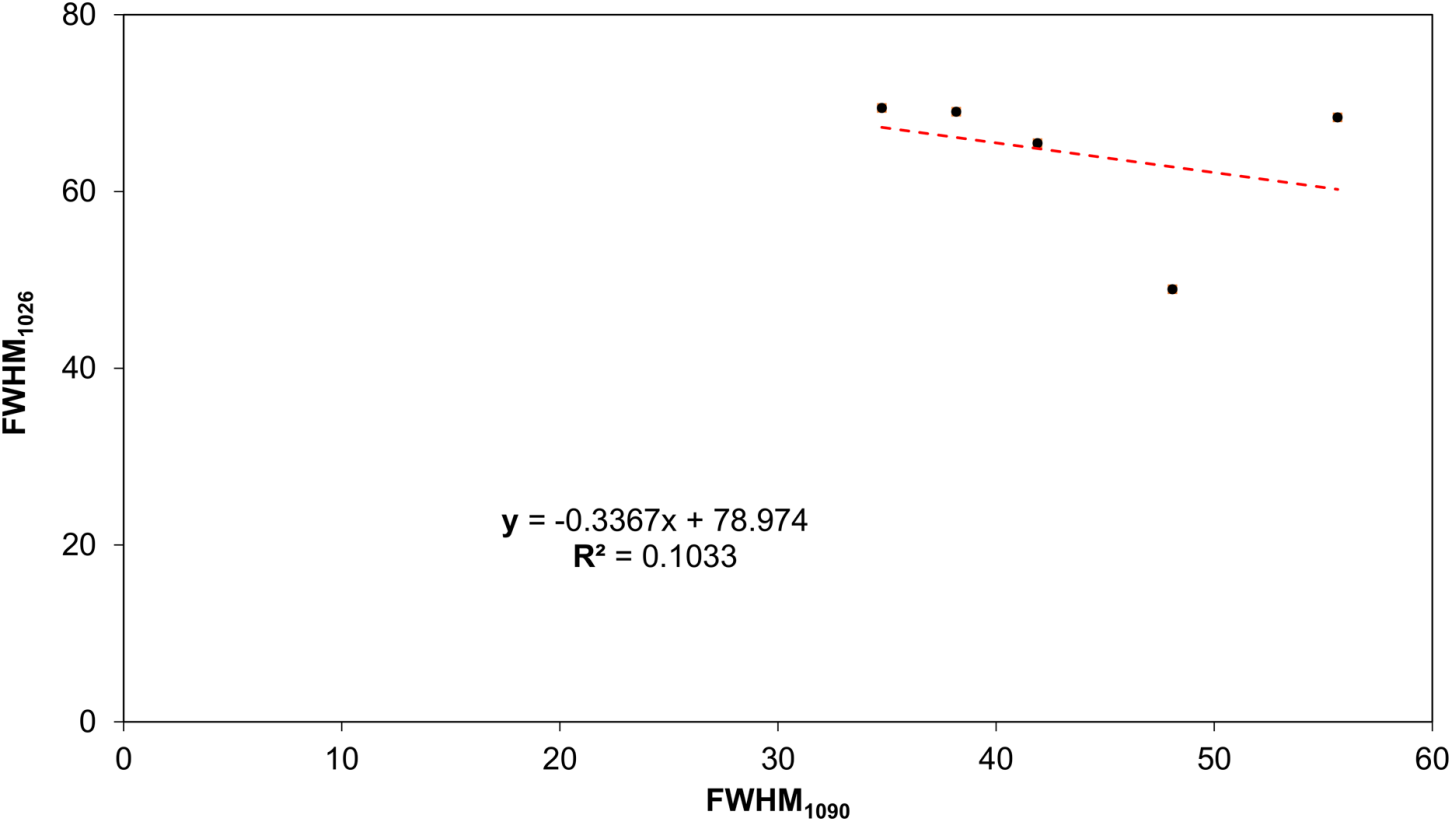
Correlation between FWHM_1090_ and FWHM_1026_, using the coefficient of determination (*R*^2^). Points indicate respectively from left to right: CTRL, G1, G2, G3 and CONV.

ATR-FTIR spectroscopy was exploited to analyze the changes in the chemical structure and composition of tooth mineralized tissues. In Fig. 7, the average IR spectra of all experimental groups are reported; three areas assigned to the organic matrix (Zone 1), to carbonate groups and, to a lesser extent, to protein side chains (Zone 2), and to phosphates (Zone 3) are showed. A similar spectral profile was found in CTRL, G1, G2 and CONV spectra, while a different shape was showed by G3 samples. Due to the presence of several convoluted bands, average IR spectra of CTRL, G1, G2, G3 and CONV groups were curve fitted in the 1,800–650 cm^−1^ spectral range based on the position of the minima of second derivative spectra, to detect all the subcomponent bands. CTRL, G1, G2 and, as further extent, CONV spectra were characterized by prominent bands related to the phosphates groups of hydroxyapatite (HA) (~1,090 cm^−1^, ~1,026 cm^−1^, ~984 cm^−1^ and ~939 cm^−1^), and to those attributable to carbonates (~1,462 cm^−1^, ~1,416 cm^−1^ and ~869 cm^−1^); weak peaks related to the organic matrix (~1,649 cm^−1^ and ~1,552 cm^−1^, Amide I and II bands of proteins) were also detected. Spectra of G3 samples were characterized by the same peaks, with an increase of the intensity of the bands attributable to the organic matrix. The statistical analysis of meaningful band area ratios was performed by Kruskal–Wallis test, followed by Dunn’s test (group size *N* = 3 for each experimental group; significance *p* < 0.05) (Fig. 8). According to the ratio A_984_+A_1090_/A_1649_ (ν1+ν3 PO_4_/Amide I), similar values (not statistically significant, *p* > 0.05) were found in CTRL, G1 and G2 groups; G3 showed a statistically significant decrease of this ratio (*p* < 0.05), while an intermediate value was observed in CONV (*p* > 0.05). A similar trend was observed in the band area ratio A_869_/A_1649_ (ν CO_3_/Amide I) (carbonates/matrix ratio), with no differences between CTRL and G1 samples (*p* > 0.05); conversely, the lowest value was found in G3 (*p* < 0.05); finally, G2 and CONV showed a small reduction of this ratio (*p* > 0.05). As regards the band area ratio A_869_/A_984_+A_1090_ (ν CO_3_/ν1+ν3 PO_4_), similar or slightly lower values were observed in CTRL, G1 and G2 groups (*p* > 0.05); also, in this case, G3 showed the lowest value (*p* < 0.05), while CONV exhibited an intermediate behavior (*p* > 0.05). In Fig. 9, the correlation between the Full Width at Half Maximum (FWHM) values of these two bands is reported. A linear correlation was found among all the experimental groups (*R*^2^ = 0.1033), with *R*^2^ improving if excluding G3 (*y* = −0.56*x* + 89.50; *R*^2^ = 0.8457). However, the presence of a negative slope, both including and excluding G3, indicates that bleaching treatments cause an increase of the amorphous hydroxyapatite.

**Figure 10:**
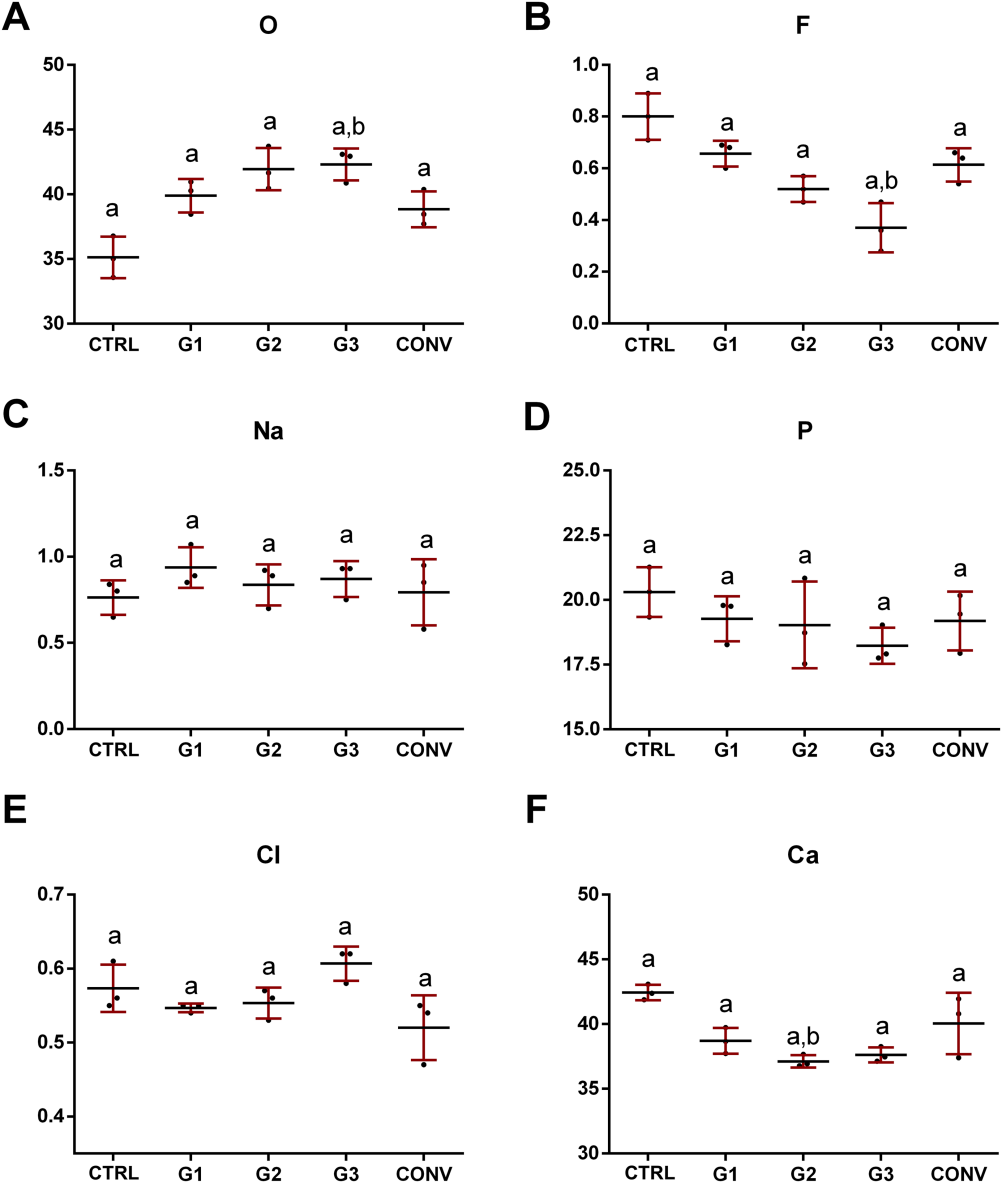
Mean values and standard deviation by weight (%) of different elements contained in the analyzed samples. (A) Oxygen, O; (B) Fluorine, F; (C) Sodium, Na; (D) Phosphorous, P; (E) Chlorine, Cl; (F) Calcium, Ca. Different lowercase letters indicate statistically significant differences among groups (*p* < 0.05).

**Figure 11:**
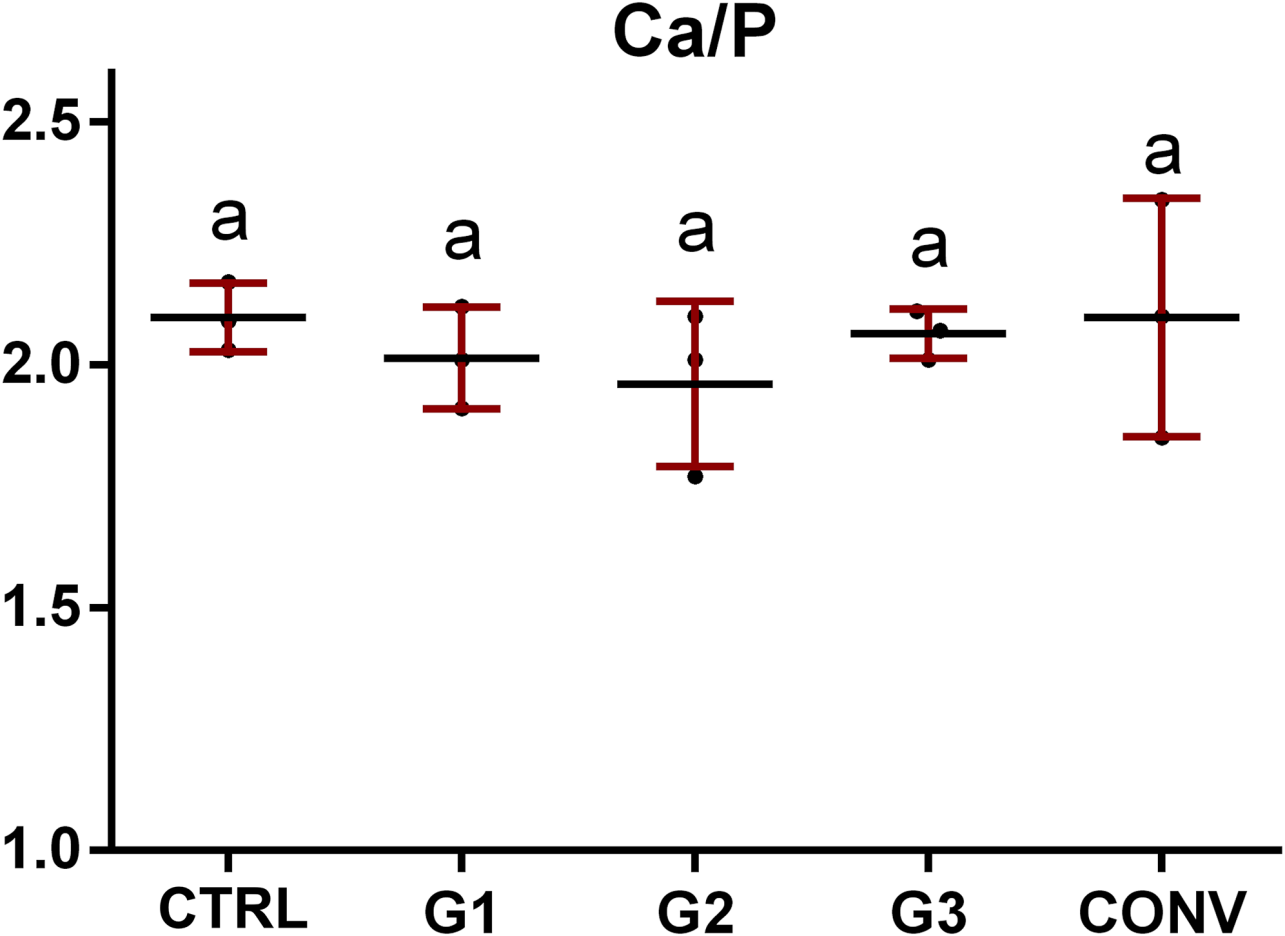
Mean values and standard deviation of Ca/P ratio, according to the treatment groups. Mean values of Ca/P ratio. Different letters indicate statistically significant differences among groups (*p* < 0.05).

For all the groups, the chemical composition of dental enamel in terms of relative amounts of O, F, Na, P, Cl and Ca elements, was analysed using EDS (Table 2). The results showed only low alterations in the levels of the following chemical constituents: (i) slightly higher levels of O in bleached samples (G1, G2, G3 and CONV) with respect to CTRL (Fig. 10A); (ii) lower levels of F in G2 and G3 samples (Fig. 10B); (iii) no statistically significant difference in Na, P, and, as further extent, Cl amounts in all groups (Figs. 10C–10E) and (iv) lower levels of Ca in G1, G2 and G3 groups (Fig. 10F). A constant ratio between Ca and P was found in all experimental groups, with a slight but not significant decrease in G2 (Fig. 11).

**Table 2:** The chemical composition of dental enamel was analyzed in all groups using EDS. The results showed low alterations in the levels of chemical constituents. Variations of the concentration values by weight (%) of the chemical elements observed by means (S.D.) of Spectroscopy of Dispersion of Ray-x (EDS) of dental enamel submitted to different bleaching agents.

	CTRL	G1	G2	G3	CONV
O	30.41 (8.18)	42.48 (4.77)	35.27 (15.64)	38.85 (5.38)	42.30 (3.27)
F	0.59 (0.36)	0.94 (1.13)	0.52 (0.49)	0.31 (0.16)	0.37 (0.10)
Na	0.60 (0.28)	0.94 (0.08)	19.03 (0.60)	19.19 (0.19)	1.00 (0.10)
P	20.81 (0.91)	18.34 (1.65)	19.03 (1.68)	19.19 (1.14)	18.23 (0.70)
Cl	0.56 (0.03)	0.50 (0.08)	0.55 (0.13)	0.52 (0.04)	0.61 (0.02)
Ca	47.03 (7.92)	36.80 (4.06)	43.78 (14.91)	40.33 (4.31)	37.49 (2.71)

## Discussion

In the last decades, the use of bleaching agents has been greatly increased. Due to this demand, a large variety of products has been developed (Pinto et al., 2014). One of the possible side-effects of bleaching treatments is that enamel structure may be weakened by the oxidation of the organic or inorganic elements. Several studies have investigated the effects of these bleaching agents in terms of mineral loss, degree of demineralization and modifications in the morphology of tooth surface, but to date, the results are quite contradictory (Shannon et al., 1993; Bitter & Sanders, 1993; Ernst, Marroquín & Willershausen-Zönnchen, 1996; Oltu & Gürgan, 2000). A study, published in 2000, examined the effects on the enamel structure of three bleaching agents containing 10%, 16% and 35% of CP (Oltu & Gürgan, 2000). The results revealed that 10% or 16% of CP did not seem to affect the structure of enamel, whereas the highest concentration caused severe damages. These results were also confirmed by a recent study, in which authors found that bleaching gel based on 10% of CP was substantially safe, but, when used for a time that exceeds that recommended by the manufacturer, could be able to reduce the micro-hardness, modify the ultrastructure and promote changes in the enamel chemical composition, only (Vilhena et al., 2019). Furthermore, Fearon et al. compared a number of studies of different whitening procedures and concluded that changes in the tooth surface structure and increased tooth sensitivity can occur, especially if highly concentrated HP and CP solutions are applied (Fearon, 2007). Moreover, some authors reported that 15% of HP is the maximum effective concentration to avoid enamel morphological alterations; higher concentrations do not improve the whitening effect, but rather increase the possibility of enamel damages. In fact, changes in the surface morphology reduces hardness properties and increases roughness (Loguercio et al., 2017; Grazioli et al., 2018; Rodríguez-Martínez, Valiente & Sánchez-Martín, 2019). Hence, in addition to the conventional bleaching materials based on various peroxide compounds, which may have some detrimental effects, scientists have been searching for alternative materials (Dabanoglu et al., 2009; Llena et al., 2019). Conversely, there is limited information discussing the use of low concentrated bleaching agents as in-office treatments and more scientific evidence should be sought on this topic. Caution is required when performing in-office bleaching, following the exact manufacturer instruction, according to the percentage and the time of application of the selected bleaching gel. Indeed, according to recent clinical studies, the current strategies to avoid the discomfort and damages associated with bleaching treatments are not totally effective, and they are not being optimized yet (Vaez et al., 2018; Rodríguez-Martínez, Valiente & Sánchez-Martín, 2019; Epple, Meyer & Enax, 2019).

This is the first in vitro study reporting ATR-FTIR, SEM, and EDS evaluation of the effects and safety of newly commercialized bleaching agents containing nHA on human third molars. The association of these techniques allows to achieve a robust correlation between imaging, spectral and chemical data, providing reliable and objective information both on the morphology and the chemical composition of the analysed samples. To improve the understanding of dental enamel structure, this approach was particularly useful, giving the opportunity to evaluate the effect of bleaching agents from different point of view.

Although the teeth were in dysodontiasis, electron micrographs of CTRL showed sound mineralized enamel surfaces without altered features, resembling the ones of normally mature post-erupted teeth (Palti et al., 2008; Markovic et al., 2010). The effects on the enamel surface of the three tested agents newly-launched on the market, with low and medium percentages of HP, loaded with nHA at different ratios were evaluated and compared with those of a 10% CP- based bleaching agent, selected for its well-documented safety and efficacy (Meireles et al., 2008; Türkün et al., 2010; Basting et al., 2012; Eachempati et al., 2018). Enamel morphological features and chemical composition of the treated groups were compared with those of a non-treated group. Findings described in this study agree with those already reported in the scientific literature. Indeed, several studies detected significant changes on the enamel surface after bleaching with concentrated 35% HP or CP, whereas 10% and 16% concentration did not alter the enamel surface (Oltu & Gürgan, 2000; Sulieman, Addy & Rees, 2003). In particular, recent studies reported that bleaching gel containing 15% of HP showed greater/neutral pH, better pH stability, lower hardness loss, and no surface damage to enamel than the 25–35% HP-based gels (Abouassi, Wolkewitz & Hahn, 2011; Magalhães et al., 2012; Grazioli et al., 2018).

Scanning Electron Microscopy analysis demonstrates that the at-home and the low percentage of the in-office bleaching agents do not seem to affect the enamel surface morphology, especially when observed at low magnification (400×). Scanning electron micrographs of CTRL teeth display the sound physiological pattern of the enamel surface, marked by the presence of the aprismatic enamel. Similarly, no significant changes in enamel structure are depicted in G1, exposed to a relatively low concentration of HP. The pattern observed in G2 is analogous to CONV, with generally common preserved features as in CTRL and G1, except in one sample which has revealed a slight increase of the enamel irregularities, with a partial loss of the interprismatic spaces and perikymata, and some microporosities. On the other hand, SEM images of G3 display a marked depth of enamel irregularities, removal of the aprismatic layer, and an evident presence of microporosities. Similar altered features have been demonstrated by other studies, in which authors concluded that gels with 25% and 35% of HP produced morphological changes on enamel, with prism rods exposure; conversely, the gel containing 15% of HP produced a similar topography to that obtained for the non-bleached specimen (Miranda et al., 2005; Kwon & Wertz, 2015; Vilhena et al., 2019). These findings are consistent with the study by Bistey et al. (2007) stating that bleaching agents based on 10–30% HP, without nHA, can provoke severe morphological alterations on the enamel surface.

Scanning Electron Microscopy results have been largely confirmed by ATR-FTIR spectroscopy. Indeed, comparable spectral profiles were found in the average IR spectra of CTRL, G1, G2 and CONV, focused on the ratio A_984_+A_1090_/A_1649_ (ν1+ν3 PO_4_/Amide I), indicated as mineral/matrix ratio, expressing the degree of mineralization of dental tissues (Boskey & Mendelsohn, 2005; Boskey et al., 2005). Conversely, G3 spectra reveal an increase of the amorphous HA and, as a further extent, of the organic matrix (Lubarsky et al., 2012). The peaks at 1,090 cm^−1^ and 1,026 cm^−1^ are usually referred to as phosphate groups in poor and well-crystallized HA (Farlay et al., 2010). The different shapes of the IR bands of phosphates of HA (Gadaleta et al., 1996), confirm the lack of mineral in enamel, as visualized by SEM microporosities.

Energy Dispersive X-ray spectroscopy is a precise and non-destructive technique for studying the mineral component of enamel (Llena, Esteve & Forner, 2018). It involves sample bombardment with a high-voltage electron beam that generates different wavelengths for each type of mineral. The changes in the wavelength of the radiation emitted by the sample indicate changes in its mineral concentration (Llena, Esteve & Forner, 2018). The obtained results revealed slight but not statistically significant differences (*p* > 0.05) in the concentration values by weight (%) of O, F and Ca elements among the experimental groups, while similar amounts were observed as regards Phosphorous and Sodium (*p* > 0.05). In particular, a slight increase in O concentration was found after the application of the tested bleaching agents. This fact is probably associated to the action of the peroxide on the mineralized enamel structure, a reaction well mentioned by the scientific literature (Cavalli, De Carvalho & Giannini, 2005; Godinho et al., 2014; Silveira et al., 2015; Vilhena et al., 2019). As regards P, a trend similar to that observed for Ca, even if not statistically significant (*p* > 0.05), was pinpointed. In fact, similar amounts of Ca were observed in CTRL and CONV (*p* > 0.05), while a slight decreasing trend was found in G1, G2 and G3 groups (*p* > 0.05), reflecting the increasing percentage of HP in the bleaching treatments. The relationship between Ca and P is an essential indicator of the remineralization process (Cakir et al., 2011; Castro et al., 2016). The Ca/P ratio calculated in this study did not show any statistically significant change in all the experimental groups (*p* > 0.05) (Vilhena et al., 2019). It is known that high concentrations of HP can modify the Ca/P ratio (Cavalli et al., 2010) and the decreases in the level of the two elements could lead to an irreversible alteration, preventing the remineralization process from occurring. All these findings suggested that the percentages of bleaching agents adopted in this study were not sufficient to cause relevant chemical alterations of the enamel (Potocnik, Kosec & Gaspersic, 2000). Therefore, according to these results, the first null hypothesis is partially rejected. As regards F values, a decreasing trend was observed when high percentages of HP are used. Similar values close to CTRL were found in G1 and CONV. G1 levels can be explained by the low percentage of HP, while those of CONV by the use of an F-containing bleaching agent. All these results suggest that low concentrated gels may be used more effectively and safely than high-concentrated agents, also for in-office bleaching procedures. Indeed, G2 seems to not significatively alter the enamel surface both qualitatively and quantitatively. On the other hand, some alterations on the enamel surface could be highlighted, as previously described for G3 (Llena, Esteve & Forner, 2017).

Existing scientific literature has well demonstrated that nHA plays a crucial role in repairing the altered enamel morphology, thanks to its major advantages as the analogy to the mineral structure of teeth, bioactivity, and biocompatibility (Vandiver et al., 2005; Pepla et al., 2014; Nozari et al., 2017). The nHA shows a strong affinity with demineralized surfaces and it is able to bind to pores created by demineralization (Li et al., 2008; Swarup & Rao, 2012). Chen et al. described the mechanism of enamel prism formation in presence of nHA. During the demineralization, nHA particles can gather together to form a stable prismatic structure such as the one observed in the enamel, thus preserving its crystallinity (Chen et al., 2005). Moreover, the remineralizing and repairing potential of nHA was also evaluated with positive outcomes in previous studies (Lelli et al., 2014; Vano et al., 2015). However, in partial disagreement with the study by Bistey et al. (2007), consistent to our results, it can be concluded that not only the 6% HP/nHA—based agent, but also the 12% HP/nHA gel did not show changes on enamel crystallinity. Indeed, SEM observations demonstrate that bleaching agents based on 6% and 12% HP, coupled with nHA, at 1:3 and 1:1, respectively, basically preserve the interprismatic enamel structure. Moreover, no changes are observed in the chemical composition of G1 and G2 when compared to CTRL. Conversely, G3 samples show an increase of the amorphous HA component of the enamel, probably ascribable to a lower amount of nHA in the bleaching gel with respect to the others. Moreover, the nHA contained in the bleaching gel used for G3 does not seem to be combined with the HA of the enamel surface, probably due to its lower pH value. Indeed, as reported by the manufacturer, the pH values of G1, G2 and G3 bleaching gels are respectively 9.2, 7.8 and 6.4, G1 and G2 showing an alkaline pH, while G3 being close to neutral. Thus it can be suggested that pH values may be relevant, as also described by several studies (Azrak et al., 2010; Basting et al., 2012; De Geus et al., 2016; Grazioli et al., 2018). CONV, containing 10% CP, is approximately equal to 3.3% HP (CP/HP ratio is 1/3) (Eachempati et al., 2018). It would be expected that a higher concentration of HP could lead to lower HA crystallinity than CONV 3.3% HP. On the contrary, our findings show that G1 and G2 have similar HA crystallinity of CONV and CTRL. Thus, the second null hypothesis can be rejected.

The absence of using saliva as a remineralizing agent after bleaching could be a limitation of our study, since saliva plays an important role in protecting the substrate from excessive mineral loss, allowing enamel remineralization (Do Amaral et al., 2012; Grazioli et al., 2018). Indeed, saliva, containing phosphate, allows to revert the effects generated by the bleaching process and to play as an alkalizing agent (Vargas-Koudriavtsev & Herrera-Sancho, 2017). However, according to other reports, since this study aims to analyze the morphology and chemical composition of the enamel surface after using bleaching agents, the teeth between each bleaching agent application were washed and immersed in distilled water in order to not alter the enamel and to not introduce a new uncontrolled variable in the research (Sulieman et al., 2004; Giulio et al., 2009; Jin et al., 2013; Vargas-Koudriavtsev & Herrera-Sancho, 2017; Farawati et al., 2019; Vilhena et al., 2019), while chloramine solution was used for their collection and maintenance (Grazioli et al., 2018; Silveira et al., 2018). Moreover, since the Ca/P ratio observed in all groups was not different from CTRL (*p* > 0.05), it can be suggested that no significant demineralization has occurred. Another limitation could be the limited range of the bleaching agents concentration analyzed, that, however, was selected among the most commonly used ones (Eachempati et al., 2018). Although some authors have claimed that microhardness test can be a reliable method to better understand the changes of the enamel physical properties (Pepla et al., 2014; Grazioli et al., 2018), even the effect of chemical alterations - as evaluated by our spectroscopic analyses - can be considered as a reliable evaluation on the physical-mechanical properties of some bleaching agents (González-López et al., 2016; Vargas-Koudriavtsev & Herrera-Sancho, 2017). Finally, further investigations might evaluate the color changes after the use of bleaching agents containing nHA and based on different HP concentrations, so as to recommend bleaching treatments that could give excellent aesthetic results without tissue damage. In this context, also clinical trials evaluating post-bleaching tooth sensitivity could be highly recommended.

## Conclusions

From this in vitro study, it can be concluded that the tested commercial bleaching agents, in particular the 10% CP and the 6% HP at-home, and the 12% HP in-office, could be safely used by dental clinicians, following the manufacturer protocol, since they neither chemically alter nor microscopically change the enamel surface. In order to better evaluate this latter topic and to improve knowledge on the mechanism of (re)action of these commercial bleaching gels on dental enamel as well as on the oral environment, it would be interesting to further propose the evaluation of these whitening agents with and without saliva interaction.

## Supplemental Information

10.7717/peerj.10606/supp-1 Supplemental Information 1 Curve fitting analysis performed on average absorbance spectra of CTRL, G1, G2, G3 and CONV groups (1800-650 cm^-1^ spectral range).

10.7717/peerj.10606/supp-2 Supplemental Information 2 Statistical test results. Exact *p* values obtained from the Tukey’s multiple comparisons test, following ANOVA, performed on EDS and ATR-FTIR results.

10.7717/peerj.10606/supp-3 Supplemental Information 3 Raw data calculated from ATR-FTIR and EDS analysis of Ctrl, G1, G2, G3 and CONV groups and used for the statistical analysis of A_984_+A_1090_/A_1649_, A_869_/A_1649_, A_869_/A_984_+A_1090_ r.

## Additional Information and Declarations

### Data Availability

The following information was supplied regarding data availability: The raw measurements are available in the Supplemental Files.

## References

[ref-1] Abouassi T, Wolkewitz M, Hahn P (2011). Effect of carbamide peroxide and hydrogen peroxide on enamel surface: an in vitro study. Clinical Oral Investigations.

[ref-2] Azrak B, Callaway A, Kurth P, Willershausen B (2010). Influence of bleaching agents on surface roughness of sound or eroded dental enamel specimens. Journal of Esthetic and Restorative Dentistry: Official Publication of the American Academy of Esthetic Dentistry.

[ref-3] Basting RT, Amaral FLB, França FMG, Flório FM (2012). Clinical comparative study of the effectiveness of and tooth sensitivity to 10% and 20% carbamide peroxide home-use and 35% and 38% hydrogen peroxide in-office bleaching materials containing desensitizing agents. Operative Dentistry.

[ref-4] Bistey T, Nagy IP, Simó A, Hegedus C (2007). In vitro FT-IR study of the effects of hydrogen peroxide on superficial tooth enamel. Journal of Dentistry.

[ref-5] Bitter NC, Sanders JL (1993). The effect of four bleaching agents on the enamel surface: a scanning electron microscopic study. Quintessence International (Berlin, Germany: 1985).

[ref-6] Bordea IR, Candrea S, Alexescu GT, Bran S, Băciuț M, Băciuț G, Lucaciu O, Dinu CM, Todea DA (2020). Nano-hydroxyapatite use in dentistry: a systematic review. Drug Metabolism Reviews.

[ref-7] Boskey AL, DiCarlo E, Paschalis E, West P, Mendelsohn R (2005). Comparison of mineral quality and quantity in iliac crest biopsies from high- and low-turnover osteoporosis: an FT-IR microspectroscopic investigation. Osteoporosis International: A Journal Established as Result of Cooperation Between the European Foundation for Osteoporosis and the National Osteoporosis Foundation of the USA.

[ref-8] Boskey AL, Mendelsohn R (2005). Infrared spectroscopic characterization of mineralized tissues. Vibrational Spectroscopy.

[ref-9] Cakir FY, Korkmaz Y, Firat E, Oztas SS, Gurgan S (2011). Chemical analysis of enamel and dentin following the application of three different at-home bleaching systems. Operative Dentistry.

[ref-10] Castro J, Godinho J, Mata A, Silveira JM, Pessanha S (2016). Study of the effects of unsupervised over-the counter whitening products on dental enamel using μ-Raman and μ-EDXRF spectroscopies. Journal of Raman Spectroscopy.

[ref-11] Cavalli V, Arrais C, Giannini M, Ambrosano G (2004). High-concentrated carbamide peroxide bleaching agents effects on enamel surface. Journal of Oral Rehabilitation.

[ref-12] Cavalli V, De Carvalho RM, Giannini M (2005). Influence of carbamide peroxide-based bleaching agents on the bond strength of resin-enamel/dentin interfaces. Brazilian Oral Research.

[ref-13] Cavalli V, Rodrigues LKA, Paes-Leme AF, Brancalion ML, Arruda MAZ, Berger SB, Giannini M (2010). Effects of bleaching agents containing fluoride and calcium on human enamel. Quintessence International (Berlin, Germany: 1985).

[ref-14] Chen H, Clarkson BH, Sun K, Mansfield JF (2005). Self-assembly of synthetic hydroxyapatite nanorods into an enamel prism-like structure. Journal of Colloid and Interface Science.

[ref-15] Coceska E, Gjorgievska E, Coleman NJ, Gabric D, Slipper IJ, Stevanovic M, Nicholson JW (2016). Enamel alteration following tooth bleaching and remineralization. Journal of Microscopy.

[ref-16] Da Costa JB, McPharlin R, Hilton T, Ferracane JI, Wang M (2012). Comparison of two at-home whitening products of similar peroxide concentration and different delivery methods. Operative Dentistry.

[ref-17] Da Silva Marques DN, Silveira JM, Marques JR, Amaral JA, Guilherme NM, Da Mata AD (2012). Kinetic release of hydrogen peroxide from different whitening products. European Journal of Esthetic Dentistry: Official Journal of the European Academy of Esthetic Dentistry.

[ref-18] Dabanoglu A, Wood C, García-Godoy F, Kunzelmann K-H (2009). Whitening effect and morphological evaluation of hydroxyapatite materials. American Journal of Dentistry.

[ref-19] Dadoun MP, Bartlett DW (2003). Safety issues when using carbamide peroxide to bleach vital teeth—a review of the literature. European Journal of Prosthodontics and Restorative Dentistry.

[ref-20] Dahl JE, Pallesen U (2003). Tooth bleaching—a critical review of the biological aspects. Critical Reviews in Oral Biology and Medicine: An Official Publication of the American Association of Oral Biologists.

[ref-21] De Geus JL, Wambier LM, Kossatz S, Loguercio AD, Reis A (2016). At-home vs in-office bleaching: a systematic review and meta-analysis. Operative Dentistry.

[ref-22] Demarco FF, Meireles SS, Masotti AS (2009). Over-the-counter whitening agents: a concise review. Brazilian Oral Research.

[ref-23] Do Amaral FLB, Sasaki RT, Da Silva TCR, França FMG, Flório FM, Basting RT (2012). The effects of home-use and in-office bleaching treatments on calcium and phosphorus concentrations in tooth enamel: an in vivo study. Journal of the American Dental Association.

[ref-24] Eachempati P, Kumbargere Nagraj S, Kiran Kumar Krishanappa S, Gupta P, Yaylali IE (2018). Home-based chemically-induced whitening (bleaching) of teeth in adults. Cochrane Database of Systematic Reviews.

[ref-25] Epple M, Meyer F, Enax J (2019). A critical review of modern concepts for teeth whitening. Dentistry Journal.

[ref-26] Ernst CP, Marroquín BB, Willershausen-Zönnchen B (1996). Effects of hydrogen peroxide-containing bleaching agents on the morphology of human enamel. Quintessence International (Berlin, Germany: 1985).

[ref-27] Farawati FAL, Hsu S-M, O’Neill E, Neal D, Clark A, Esquivel-Upshaw J (2019). Effect of carbamide peroxide bleaching on enamel characteristics and susceptibility to further discoloration. Journal of Prosthetic Dentistry.

[ref-28] Farlay D, Panczer G, Rey C, Delmas PD, Boivin G (2010). Mineral maturity and crystallinity index are distinct characteristics of bone mineral. Journal of Bone and Mineral Metabolism.

[ref-29] Fearon J (2007). Tooth whitening: concepts and controversies. Journal of the Irish Dental Association.

[ref-30] Furlan IS, Bridi EC, Do Amaral FLB, França FMG, Turssi CP, Basting RT (2017). Effect of high- or low-concentration bleaching agents containing calcium and/or fluoride on enamel microhardness. General Dentistry.

[ref-31] Gadaleta SJ, Paschalis EP, Betts F, Mendelsohn R, Boskey AL (1996). Fourier transform infrared spectroscopy of the solution-mediated conversion of amorphous calcium phosphate to hydroxyapatite: new correlations between X-ray diffraction and infrared data. Calcified Tissue International.

[ref-32] Giulio AB, Matteo Z, Serena IP, Silvia M, Luigi C (2009). In vitro evaluation of casein phosphopeptide-amorphous calcium phosphate (CPP-ACP) effect on stripped enamel surfaces—a SEM investigation. Journal of Dentistry.

[ref-33] Godinho J, Silveira J, Mata A, Carvalho ML, Pessanha S (2014). Effect of bleaching gel in Ca, P and Zn content in tooth enamel evaluated by μ-EDXRF. Nuclear Instruments and Methods in Physics Research Section B: Beam Interactions with Materials and Atoms.

[ref-34] González-López S, Torres-Rodríguez C, Bolaños-Carmona V, Sanchez-Sanchez P, Rodríguez-Navarro A, Álvarez-Lloret P, Domingo Garcia M (2016). Effect of 30% hydrogen peroxide on mineral chemical composition and surface morphology of bovine enamel. Odontology.

[ref-35] Grazioli G, Valente LL, Isolan CP, Pinheiro HA, Duarte CG, Münchow EA (2018). Bleaching and enamel surface interactions resulting from the use of highly-concentrated bleaching gels. Archives of Oral Biology.

[ref-36] Huang SB, Gao SS, Yu HY (2009). Effect of nano-hydroxyapatite concentration on remineralization of initial enamel lesion in vitro. Biomedical Materials.

[ref-37] Jin J, Xu X, Lai G, Kunzelmann K-H (2013). Efficacy of tooth whitening with different calcium phosphate-based formulations. European Journal of Oral Sciences.

[ref-38] Joiner A (2010). Whitening toothpastes: a review of the literature. Journal of Dentistry.

[ref-39] Joiner A, Hopkinson I, Deng Y, Westland S (2008). A review of tooth colour and whiteness. Journal of Dentistry.

[ref-40] Kawamoto K, Tsujimoto Y (2004). Effects of the hydroxyl radical and hydrogen peroxide on tooth bleaching. Journal of Endodontics.

[ref-41] Kowalczuk D, Pitucha M (2019). Application of FTIR method for the assessment of immobilization of active substances in the matrix of biomedical materials. Materials.

[ref-42] Kutuk ZB, Ergin E, Cakir FY, Gurgan S (2018). Effects of in-office bleaching agent combined with different desensitizing agents on enamel. Journal of Applied Oral Science: Revista FOB.

[ref-43] Kwon SR, Wertz PW (2015). Review of the mechanism of tooth whitening. Journal of Esthetic and Restorative Dentistry: Official Publication of the American Academy of Esthetic Dentistry.

[ref-44] Lelli M, Putignano A, Marchetti M, Foltran I, Mangani F, Procaccini M, Roveri N, Orsini G (2014). Remineralization and repair of enamel surface by biomimetic Zn-carbonate hydroxyapatite containing toothpaste: a comparative in vivo study. Frontiers in Physiology.

[ref-45] Li L, Pan H, Tao J, Xu X, Mao C, Gu X, Tang R (2008). Repair of enamel by using hydroxyapatite nanoparticles as the building blocks. Journal of Materials Chemistry.

[ref-46] Liu Y, Yao X, Liu Y, Wang Y (2014). A fourier transform infrared spectroscopy analysis of carious dentin from transparent zone to normal zone. Caries Research.

[ref-47] Llena C, Esteve I, Forner L (2017). Effect of hydrogen and carbamide peroxide in bleaching, enamel morphology, and mineral composition: in vitro study. Journal of Contemporary Dental Practice.

[ref-48] Llena C, Esteve I, Forner L (2018). Effects of in-office bleaching on human enamel and dentin—morphological and mineral changes: annals of anatomy = anatomischer anzeiger. Official Organ of the Anatomische Gesellschaft.

[ref-49] Llena C, Esteve I, Rodríguez-Lozano FJ, Forner L (2019). The application of casein phosphopeptide and amorphous calcium phosphate with fluoride (CPP-ACPF) for restoring mineral loss after dental bleaching with hydrogen or carbamide peroxide: an in vitro study. Annals of Anatomy—Anatomischer Anzeiger.

[ref-50] Loguercio AD, Servat F, Stanislawczuk R, Mena-Serrano A, Rezende M, Prieto MV, Cereño V, Rojas MF, Ortega K, Fernandez E, Reis A (2017). Effect of acidity of in-office bleaching gels on tooth sensitivity and whitening: a two-center double-blind randomized clinical trial. Clinical Oral Investigations.

[ref-51] Lubarsky GV, D’Sa RA, Deb S, Meenan BJ, Lemoine P (2012). The role of enamel proteins in protecting mature human enamel against acidic environments: a double layer force spectroscopy study. Biointerphases.

[ref-52] Magalhães JG, Marimoto ARK, Torres CRG, Pagani C, Teixeira SC, Barcellos DC (2012). Microhardness change of enamel due to bleaching with in-office bleaching gels of different acidity. Acta Odontologica Scandinavica.

[ref-53] Markovic L, Fotouhi K, Lorenz H, Jordan RA, Gaengler P, Zimmer S (2010). Effects of bleaching agents on human enamel light reflectance. Operative Dentistry.

[ref-54] Meireles SS, Heckmann SS, Leida FL, Dos Santos I da S, Della Bona A, Demarco FF (2008). Efficacy and safety of 10% and 16% carbamide peroxide tooth-whitening gels: a randomized clinical trial. Operative Dentistry.

[ref-55] Miranda CB, Pagani C, Benetti AR, Da Matuda FS (2005). Evaluation of the bleached human enamel by scanning electron microscopy. Journal of Applied Oral Science: Revista FOB.

[ref-56] Nozari A, Ajami S, Rafiei A, Niazi E (2017). Impact of nano hydroxyapatite, nano silver fluoride and sodium fluoride varnish on primary teeth enamel remineralization: an in vitro study. Journal of Clinical and Diagnostic Research: JCDR.

[ref-57] Oltu U, Gürgan S (2000). Effects of three concentrations of carbamide peroxide on the structure of enamel. Journal of Oral Rehabilitation.

[ref-58] Orilisi G, Monterubbianesi R, Tosco V, Conti C, Procaccini M, Putignano A, Orsini G (2020). Effect of a sodium fluoride-releasing rubber cup on hydroxyapatite crystallinity of human enamel: FTIR spectroscopy analysis. Dental Research and Oral Health.

[ref-59] Palti DG, De Machado AMMA, Da Silva SMB, Abdo RCC, De Lima OJE (2008). Evaluation of superficial microhardness in dental enamel with different eruptive ages. Brazilian Oral Research.

[ref-60] Paschalis EP, Mendelsohn R, Boskey AL (2011). Infrared assessment of bone quality: a review. Clinical Orthopaedics and Related Research.

[ref-61] Pepla E, Besharat LK, Palaia G, Tenore G, Migliau G (2014). Nano-hydroxyapatite and its applications in preventive, restorative and regenerative dentistry: a review of literature. Annali di Stomatologia.

[ref-62] Perdigão J, Baratieri LN, Arcari GM (2004). Contemporary trends and techniques in tooth whitening: a review. Practical Procedures & Aesthetic Dentistry: PPAD.

[ref-63] Pintado-Palomino K, Peitl Filho O, Zanotto ED, Tirapelli C (2015). A clinical, randomized, controlled study on the use of desensitizing agents during tooth bleaching. Journal of Dentistry.

[ref-64] Pinto MM, De Godoy CHL, Bortoletto CC, Olivan SRG, Motta LJ, Altavista OM, Lumi K, Sobral APT, Bussadori SK (2014). Tooth whitening with hydrogen peroxide in adolescents: study protocol for a randomized controlled trial. Trials.

[ref-65] Potocnik I, Kosec L, Gaspersic D (2000). Effect of 10% carbamide peroxide bleaching gel on enamel microhardness, microstructure, and mineral content. Journal of Endodontics.

[ref-66] Rodríguez-Martínez J, Valiente M, Sánchez-Martín M-J (2019). Tooth whitening: from the established treatments to novel approaches to prevent side effects. Journal of Esthetic and Restorative Dentistry.

[ref-67] Shannon H, Spencer P, Gross K, Tira D (1993). Characterization of enamel exposed to 10% carbamide peroxide bleaching agents. Quintessence International (Berlin, Germany: 1985).

[ref-68] Sherwood IA (2010). Fluorosis varied treatment options. Journal of Conservative Dentistry: JCD.

[ref-69] Shi X-C, Ma H, Zhou J-L, Li W (2012). The effect of cold-light-activated bleaching treatment on enamel surfaces in vitro. International Journal of Oral Science.

[ref-70] Silveira J, Coutinho S, Marques D, Castro J, Mata A, Carvalho ML, Pessanha S (2018). Raman spectroscopy analysis of dental enamel treated with whitening product—influence of saliva in the remineralization. Spectrochimica Acta—Part A, Molecular and Biomolecular Spectroscopy.

[ref-71] Silveira J, Godinho J, Mata A, Carvalho ML, Pessanha S (2015). Assessment of teeth elemental content using μ-EDXRF: effects by in-office and at-home bleaching products. X-ray Spectrometry.

[ref-72] Sulieman M, Addy M, MacDonald E, Rees JS (2004). The effect of hydrogen peroxide concentration on the outcome of tooth whitening: an in vitro study. Journal of Dentistry.

[ref-73] Sulieman M, Addy M, Rees JS (2003). Development and evaluation of a method in vitro to study the effectiveness of tooth bleaching. Journal of Dentistry.

[ref-74] Swarup JS, Rao A (2012). Enamel surface remineralization: using synthetic nanohydroxyapatite. Contemporary Clinical Dentistry.

[ref-75] Türkün M, Celik EU, Aladağ A, Gökay N (2010). One-year clinical evaluation of the efficacy of a new daytime at-home bleaching technique. Journal of Esthetic and Restorative Dentistry: Official Publication of the American Academy of Esthetic Dentistry.

[ref-76] Vaez SC, Faria-e-Silva AL, Loguércio AD, Fernandes MTG, Nahsan FPS (2018). Preemptive use of etodolac on tooth sensitivity after in-office bleaching: a randomized clinical trial. Journal of Applied Oral Science.

[ref-77] Vandiver J, Dean D, Patel N, Bonfield W, Ortiz C (2005). Nanoscale variation in surface charge of synthetic hydroxyapatite detected by chemically and spatially specific high-resolution force spectroscopy. Biomaterials.

[ref-78] Vano M, Derchi G, Barone A, Genovesi A, Covani U (2015). Tooth bleaching with hydrogen peroxide and nano-hydroxyapatite: a 9-month follow-up randomized clinical trial. International Journal of Dental Hygiene.

[ref-79] Vargas-Koudriavtsev T, Herrera-Sancho ÓA (2017). Effect of tooth-bleaching on the carbonate concentration in dental enamel by Raman spectroscopy. Journal of Clinical and Experimental Dentistry.

[ref-80] Verdelis K, Lukashova L, Wright JT, Mendelsohn R, Peterson MGE, Doty S, Boskey AL (2007). Maturational changes in dentin mineral properties. Bone.

[ref-81] Vilhena KFB, Nogueira BCL, Fagundes NCF, Loretto SC, Angelica RS, Lima RR, Silva e Souza MH (2019). Dental enamel bleached for a prolonged and excessive time: morphological changes. PLOS ONE.

[ref-82] Wang W, Zhu Y, Li J, Liao S, Ai H (2013). Efficacy of cold light bleaching using different bleaching times and their effects on human enamel. Dental Materials Journal.

[ref-83] Watts A, Addy M (2001). Tooth discolouration and staining: a review of the literature. British Dental Journal.

