# Supplementary figures and images for: ATR-FTIR, EDS and SEM evaluations of enamel structure after treatment with hydrogen peroxide bleaching agents loaded with nano-hydroxyapatite particles
Source: PeerJ. 2021 Jan 29;9:e10606. doi: 10.7717/peerj.10606 (PMC7849511; doi:10.7717/peerj.10606)

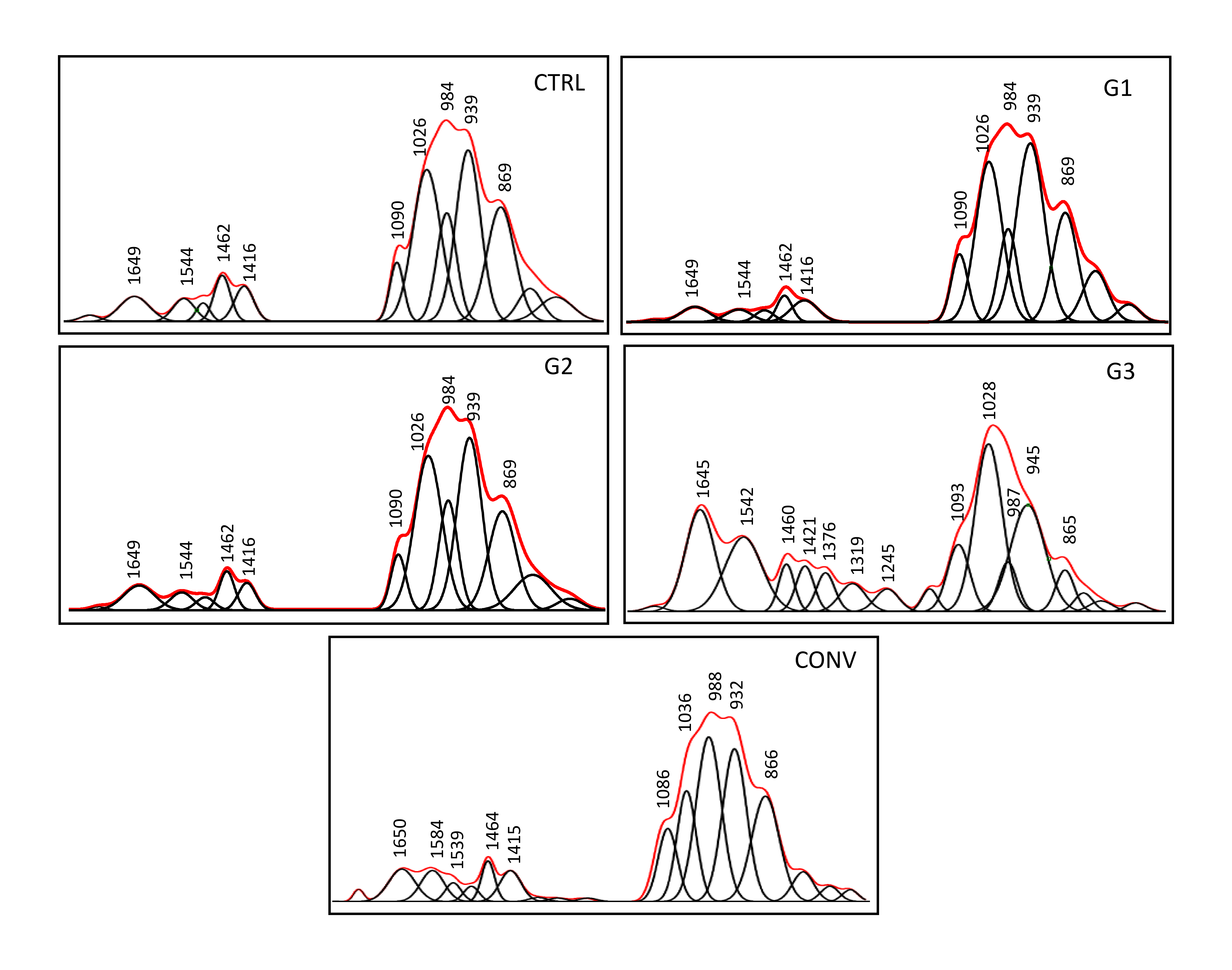

Supplement: Supplemental Information 1 [file peerj-09-10606-s001.png]
