# Supplementary material for: ATR-FTIR, EDS and SEM evaluations of enamel structure after treatment with hydrogen peroxide bleaching agents loaded with nano-hydroxyapatite particles
Source: PeerJ. 2021 Jan 29;9:e10606. doi: 10.7717/peerj.10606 (PMC7849511; doi:10.7717/peerj.10606)
Supplement: Supplemental Information 2 — Exact p values obtained from the Tukey’s multiple comparisons test, following ANOVA, performed on EDS and ATR-FTIR results. [file peerj-09-10606-s002.docx]

SUPPLEMENTARY MATERIAL

Table S1. Adjusted p values obtained from the Dunn’s multiple comparisons test, following Kruskal-Wallis test, performed on relative amounts of O, F, Na, P, Cl and Ca elements, measured by EDS for Ctrl, G1, G2, G3 and CONV.

|  | ***Exact p values*** | | | | | |
| --- | --- | --- | --- | --- | --- | --- |
|  | **O** | **F** | **Na** | **P** | **Cl** | **Ca** |
| Ctrl vs. G1 | >0.9999 | >0.9999 | 0.8208 | >0.9999 | >0.9999 | >0.9999 |
| Ctrl vs. G2 | 0.0619 | 0.1550 | >0.9999 | >0.9999 | >0.9999 | 0.0350 |
| Ctrl vs. G3 | 0.0467 | 0.0119 | >0.9999 | 0.2851 | >0.9999 | 0.2252 |
| Ctrl vs. CONV | >0.9999 | >0.9999 | >0.9999 | >0.9999 | 0.9646 | >0.9999 |
| G1 vs. G2 | >0.9999 | >0.9999 | >0.9999 | >0.9999 | >0.9999 | >0.9999 |
| G1 vs. G3 | >0.9999 | 0.2523 | >0.9999 | >0.9999 | 0.2368 | >0.9999 |
| G1 vs. CONV | >0.9999 | >0.9999 | >0.9999 | >0.9999 | >0.9999 | >0.9999 |
| G2 vs. G3 | >0.9999 | >0.9999 | >0.9999 | >0.9999 | 0.8756 | >0.9999 |
| G2 vs. CONV | >0.9999 | >0.9999 | >0.9999 | >0.9999 | >0.9999 | 0.8290 |
| G3 vs. CONV | 0.8290 | 0.9103 | >0.9999 | >0.9999 | 0.0561 | >0.9999 |

Table S2. Adjusted p values obtained from the Dunn’s multiple comparisons test, following Kruskal-Wallis test, performed on the values of the ratios A_984_+A_1090_/A_1649_, A_869_/A_1649_, and A_869_/A_984_+A_1090_, calculated for Ctrl, G1, G2, G3 and CONV.

|  | ***Exact p values*** | | |
| --- | --- | --- | --- |
|  | **A_984_+A_1090_/A_1649_** | **A_869_/A_1649_** | **A_869_/A_984_+A_1090_** |
| Ctrl vs. G1 | >0.9999 | >0.9999 | >0.9999 |
| Ctrl vs. G2 | >0.9999 | >0.9999 | >0.9999 |
| Ctrl vs. G3 | 0.0196 | 0.0196 | 0.0453 |
| Ctrl vs. CONV | 0.2018 | 0.2018 | 0.3892 |
| G1 vs. G2 | >0.9999 | >0.9999 | >0.9999 |
| G1 vs. G3 | 0.5286 | 0.2018 | 0.0985 |
| G1 vs. CONV | >0.9999 | >0.9999 | 0.7076 |
| G2 vs. G3 | 0.5286 | >0.9999 | >0.9999 |
| G2 vs. CONV | >0.9999 | >0.9999 | >0.9999 |
| G3 vs. CONV | >0.9999 | >0.9999 | >0.9999 |
